# New Modified Exponentiated Weibull Distribution: A Survival Analysis

**DOI:** 10.7759/cureus.77347

**Published:** 2025-01-12

**Authors:** A M Rangoli, A S Talawar, R P Agadi, Vijaya Sorganvi

**Affiliations:** 1 Department of Community Medicine, Shri B M Patil Medical College Hospital and Research Centre, Vijayapura, IND; 2 Department of Statistics, Karnatak University Dharwad, Dharwad, IND; 3 Department of Community Medicine, Shridevi Institute of Medical Sciences and Research Hospital, Tumakuru, IND

**Keywords:** em algorithm, hazard, information criterion, kaplan-meier survival curves, modified weibull distribution, survival

## Abstract

Introduction

In survival analysis, various lifetime distributions are used to model hazard and survival functions. This study introduces a modified Weibull distribution capable of exhibiting increasing, decreasing, constant, and bathtub-shaped hazard rates. The distribution's flexibility allows it to better capture different density patterns like bimodal observed in real-world data, especially in medical settings.

Methodology

The proposed distribution's properties, including its hazard and survival functions, are explored in detail. Data from hospital records was used to validate the model. Parameters are estimated via the expectation-maximization (EM) algorithm, with standard errors and confidence intervals calculated. A comparison is drawn with other modified Weibull models to assess the performance.

Results

The model demonstrates a good fit for the hospital dataset, providing a robust estimation of survival probabilities across different time periods. The EM algorithm ensures precise parameter estimation, and the results show the model’s capability to capture varying hazard patterns effectively. Kaplan-Meier survival curves are plotted and compared with the survival curve from the proposed model, showing strong alignment.

Conclusion

The modified Weibull distribution introduced in this study offers a versatile tool for modeling different hazard rate patterns. The model's strong performance, validated through real hospital data, suggests it could be a valuable addition to survival analysis, outperforming other modified Weibull models in terms of fit and flexibility.

## Introduction

In recent years, there has been a surge in interest in survival analysis, driven by the increasing complexity of survival data in clinical trials, public health studies, and industrial reliability assessments. These trends emphasize the need for accurate models of survival probabilities, which may decline over time in certain contexts. By incorporating time-to-event data and censoring, survival analysis offers a robust framework for analyzing factors that influence survival duration across populations.

Accurate modeling of failure rates is crucial, requiring life distributions that capture the characteristics of these rates. Over the years, various life distributions have been developed or modified to account for different hazard rate patterns. Among these, the Weibull distribution (WD) is widely recognized in reliability analysis due to its flexibility in modeling diverse failure rate behaviors. However, to better accommodate the complexities of real-world data, researchers have introduced extensions, exponentiated versions, and modifications of the Weibull distribution. These modifications introduce additional parameters to capture a broader range of failure behaviors, as seen in studies [1-11].

In this paper, we propose a new modified Weibull distribution with four parameters and estimate its hazard and survival functions. This new distribution is particularly useful for modeling bimodal densities and non-monotonic hazard functions.

## Materials and methods

Data description

Data was collected from Shri B. M. Patil Medical Hospital and Research Center in Vijayapura, India, detailing the failure times of patients admitted during the study period. The data spans from January 1, 2023, to December 31, 2023, and includes 936 patients who died due to various causes during this time frame.

Model Formulation

Let T_1_, T_2_,….,T_n_ are failure times, we assume that T_i_ to follows modified Weibull random variables with parameters (α, β, γ, λ), i=1,2,…n.

Let f(t), F(t), S(t) and λ(t) be density, cumulative distribution, survival and hazard functions of the new modified Weibull distribution.

Let the cumulative distribution function (CDF) is, 

$F(t) = 1 - e^{- \left( \frac{\alpha}{\beta^{\alpha}}\left( e^{t^{\beta}} - 1 \right) + \gamma t^{\lambda} \right)} \tag{ (1)}$

where α > 0 ,β > 0 , γ ≥ 0, λ > 0, t ≥ 0, β and λ are shape parameters, α and γ are scale parameters.

The probability density function (PDF) is, 

$f(t) = \left( \frac{\alpha}{\beta^{\alpha}} \beta t^{\beta - 1} e^{t^{\beta}} + \gamma \lambda t^{\lambda - 1} \right) e^{-\left( \frac{\alpha}{\beta^{\alpha}} \left( e^{t^{\beta}} - 1 \right) + \gamma t^{\lambda} \right)} \tag{ (2)}$

Hazard function is, 

$\lambda(t) = \left( \frac{\alpha}{\beta^{\alpha}} \beta t^{\beta - 1} e^{t^{\beta}} + \gamma \lambda t^{\lambda - 1} \right) \tag{ (3)}$

Survival function is, 

$S(t) = e^{- \left( \frac{\alpha}{\beta^{\alpha}}\left( e^{t^{\beta}} - 1 \right) + \gamma t^{\lambda} \right)} \tag{ (4)}$

This modified Weibull distribution is widely used in various fields such as reliability analysis, human mortality studies, and survival analysis. Its versatility in modeling different types of failure patterns and hazard rates makes it an invaluable tool for understanding time-to-event data in both industrial and healthcare contexts.

Motivation for the Modified Weibull Distribution

**Figure 1:**
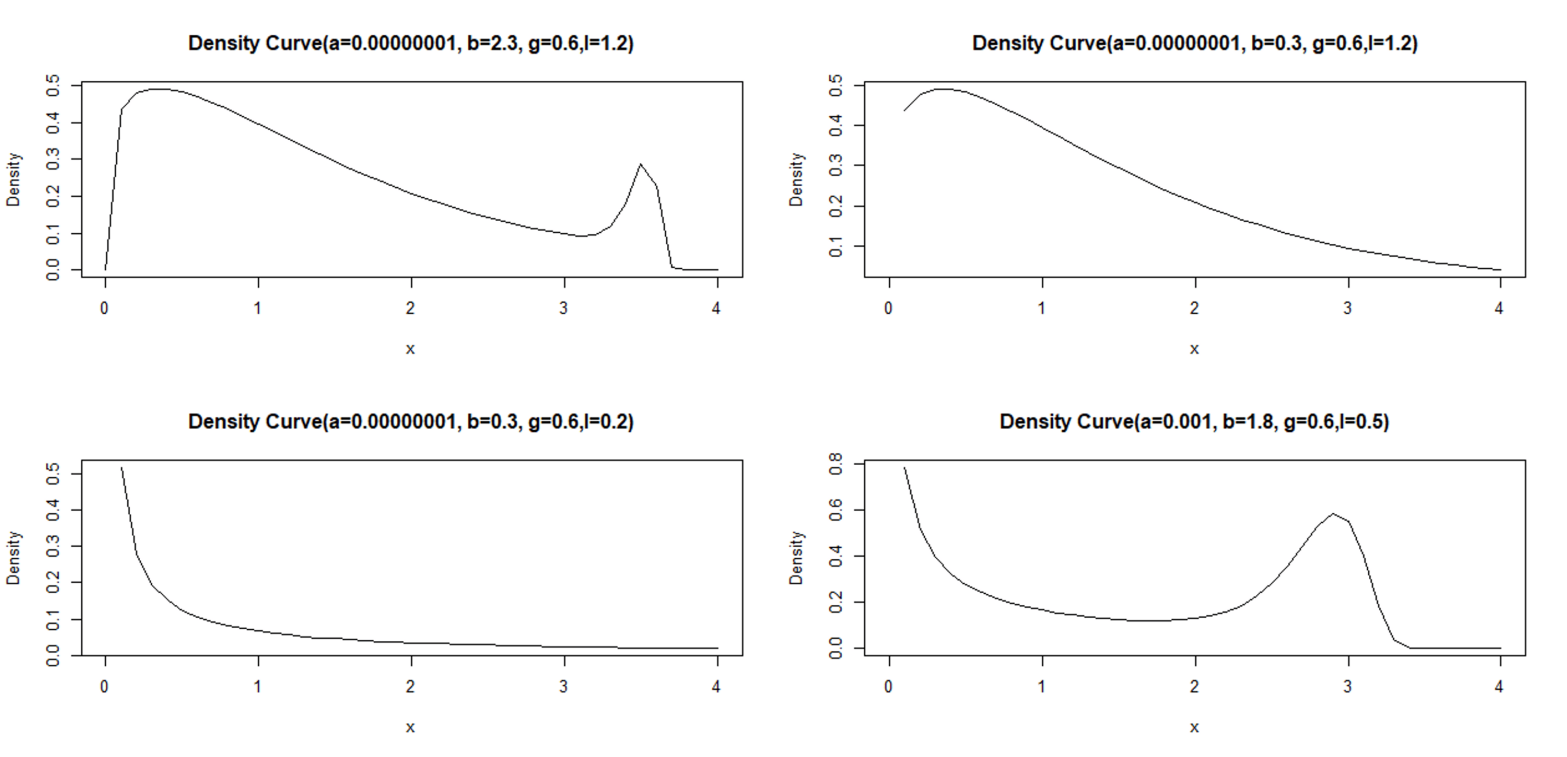
Density curves for different values of parameters.

**Figure 2:**
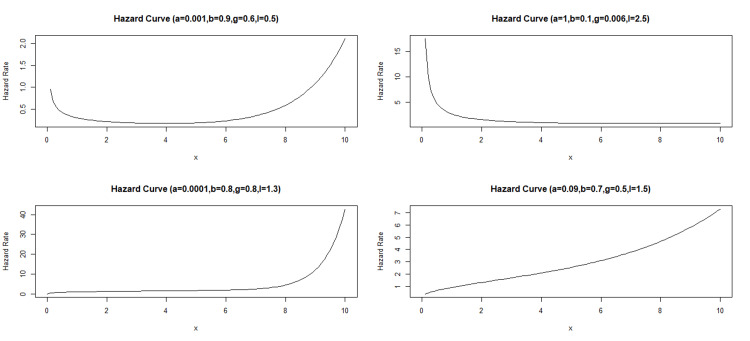
Hazard curves for different values of parameters.

Here are two key motivations for the proposed modified Weibull distribution with four parameters: the proposed distribution offers a wide range of shapes, including both unimodal and bimodal density patterns (Figure 1), making it highly applicable to real-life scenarios; the new probabilistic model exhibits various hazard rate function shapes, such as bathtub, decreasing, constant-increasing, and increasing hazard rates (Figure 2). This flexibility makes the modified Weibull distribution particularly useful for fitting diverse datasets with different hazard rate shapes.

## Results

Some properties

Mean

The mean of the random variable T with pdf f(t) is given by,

$E(T) = \int_{0}^{\infty} t f(t)\, dt$

$E(T) = \int_{0}^{\infty} t \left( \frac{\alpha}{\beta^{\alpha}} \beta t^{\beta - 1} e^{t^{\beta}} + \gamma \lambda t^{\lambda - 1} \right) e^{- \left( \frac{\alpha}{\beta^{\alpha}} \left( e^{t^{\beta}} - 1 \right) + \gamma t^{\lambda} \right)} \, dt$

This integral does not have a simple closed form, so numerical method should be used to estimate mean. The mean represents the average time until the event occurs.

Variance

The variance measures the spread of the time to failure.

$V(T) = E\left( T^{2} \right) - \left( E(T) \right)^{2}$

$E\left( T^{2} \right) = \int_{0}^{\infty} t^{2} f(t)\, dt$

$E\left( T^{2} \right) = \int_{0}^{\infty} t^{2} \left( \frac{\alpha}{\beta^{\alpha}} \beta t^{\beta - 1} e^{t^{\beta}} + \gamma \lambda t^{\lambda - 1} \right) e^{- \left( \frac{\alpha}{\beta^{\alpha}} \left( e^{t^{\beta}} - 1 \right) + \gamma t^{\lambda} \right)} \, dt$

This is also computed using numerical method.

Moment Generating Function (MGF)

The moment is given by, $E\left( T^{r} \right)$ that is, $E\left( T^{r} \right) = \int_{0}^{\infty} t^{r} f(t) \, dt$

The MGF is defined as, $E\left( T^{r} \right) = \int_{0}^{\infty} t^{r} f(t) \, dt$

$M_{T}(s) = \int_{0}^{\infty} \left( \frac{\alpha}{\beta^{\alpha}} \beta t^{\beta - 1} e^{t^{\beta}} + \gamma \lambda t^{\lambda - 1} \right) e^{sT - \left( \frac{\alpha}{\beta^{\alpha}} \left( e^{t^{\beta}} - 1 \right) + \gamma t^{\lambda} \right)} \, dt$

The MGF provides a way to compute all moments of the distribution by taking derivates,

$E\left( T^{r} \right) = \left. \frac{d^{r} M_{T}(s)}{ds^{r}} \right|_{s = 0}$

Here if r=1 we get first moment (Mean, E(T)), and r=2 we get second moment (E(T^2^ )).

Cumulative Hazard Function

The cumulative hazard function is given by, 

$H(t) = \int_{0}^{t} \lambda(u) \, du = \int_{0}^{t} \left( \frac{\alpha}{\beta^{\alpha}} \beta u^{\beta - 1} e^{u^{\beta}} + \gamma \lambda u^{\lambda - 1} \right) \, du$

$H(t) = \frac{\alpha}{\beta^{\alpha}} \beta \int_{0}^{t} u^{\beta - 1} e^{u^{\beta}} \, du + \gamma \lambda \int_{0}^{t} u^{\lambda - 1} \, du$

$H(t) = \frac{\alpha}{\beta^{\alpha}} \left( e^{t^{\beta}} - 1 \right) + \gamma t^{\lambda}$, so we write $S(t) = e^{-H(t)}$.

Quantile Function

The quantile function is the inverse of the CDF. For a given probability p the quantile function Q(p) gives the value at t such that probability of failure by that time is p,

$Q(p) = F^{-1}(p)$

$F(t) = p$

$1 - e^{- \left( \frac{\alpha}{\beta^{\alpha}} \left( e^{t^{\beta}} - 1 \right) + \gamma t^{\lambda} \right)} = p$

$\frac{\alpha}{\beta^{\alpha}} \left( e^{t^{\beta}} - 1 \right) + \gamma t^{\lambda} = -\ln(1 - p)$

This equation can besolved using numerical method. If we substitute p=0.25, 0.5, 0.75, we get first, second (median) and third quartiles respectively.

Order Statistics

Let T_1_, T_2_,….,T_n_ be a random sample of size n from modified Weibull distribution with PDF and CDF defined in (2) and (1) respectively. We give the density of r^th^ order statistic as, 

$f_{r}(t) = \frac{n!}{(r - 1)!(n - r)!} \left( 1 - e^{- \left( \frac{\alpha}{\beta^{\alpha}} \left( e^{t^{\beta}} - 1 \right) + \gamma t^{\lambda} \right)} \right)^{r - 1} \left( e^{- \left( \frac{\alpha}{\beta^{\alpha}} \left( e^{t^{\beta}} - 1 \right) + \gamma t^{\lambda} \right)} \right)^{n - r + 1} \left( \frac{\alpha}{\beta^{\alpha}} \beta t^{\beta - 1} e^{t^{\beta}} + \gamma \lambda t^{\lambda - 1} \right)$

The density of I^st^ order statistic is given by, 

$f_{1}(t) = n \left( e^{- \left( \frac{\alpha}{\beta^{\alpha}} \left( e^{t^{\beta}} - 1 \right) + \gamma t^{\lambda} \right)} \right)^{n} \left( \frac{\alpha}{\beta^{\alpha}} \beta t^{\beta - 1} e^{t^{\beta}} + \gamma \lambda t^{\lambda - 1} \right)$

And the density of n^_th_^ order statistic is given by, 

$f_{n}(t) = n \left( 1 - e^{- \left( \frac{\alpha}{\beta^{\alpha}} \left( e^{t^{\beta}} - 1 \right) + \gamma t^{\lambda} \right)} \right)^{n - 1} \left( \frac{\alpha}{\beta^{\alpha}} \beta t^{\beta - 1} e^{t^{\beta}} + \gamma \lambda t^{\lambda - 1} \right) e^{- \left( \frac{\alpha}{\beta^{\alpha}} \left( e^{t^{\beta}} - 1 \right) + \gamma t^{\lambda} \right)}$

Parameter estimation

Maximum Likelihood Estimation

The maximum likelihood estimation (MLE) is widely used among the statistical inference because of its desirable properties like consistency, asymptotic efficiency. Use of maximum likelihood estimation is illustrated below.

The likelihood function is given by, $L = \prod_{i = 1}^{n} f(t_{i}) \tag{5}$

The log likelihood can be written as, 

$\log L = \sum_{i = 1}^{n} \log\left( f\left( t_{i} \right) \right)$

$\log L = \sum_{i = 1}^{n} \log{\left( \frac{\alpha}{\beta^{\alpha}} \beta t^{\beta - 1} e^{t^{\beta}} + \gamma \lambda t^{\lambda - 1} \right)} - \sum_{i = 1}^{n} \left( \frac{\alpha}{\beta^{\alpha}} \left( e^{t^{\beta}} - 1 \right) + \gamma t^{\lambda} \right) \tag{6}$

By partially differentiating equation (6) with respect to each parameter and setting the resulting expressions to zero, we obtain the parameter estimates. The first-order partial derivatives are provided in Appendix 1. However, since the partial differentiation does not yield an explicit solution, we consider using either a numerical method, such as the Newton-Raphson method, or the expectation-maximization (EM) algorithm.

Expectation-Maximization (EM) Algorithm

The expectation-maximization (EM) algorithm is a widely used iterative method for finding maximum likelihood estimates of parameters in probabilistic models, especially when the data is incomplete or has missing values [12]. Here’s how the EM algorithm works:

Steps of the EM Algorithm

Initialization: Start with initial guesses for the parameters $\hat{\theta}^{(0)}$

Expectation step (E-step): Compute the expected value of the log-likelihood function, with respect to the unknown (latent) variables, using the current estimate of the parameters $\hat{\theta}^{(t)}$. This step calculates the "responsibilities" or the probability of the latent variables, given the observed data and current parameters.

Formally, this is the expected value of the complete-data log-likelihood

$Q(\theta \mid \hat{\theta}^{(t)}) = \mathbb{E}\left[\log p(X, Z \mid \theta) \mid X, \hat{\theta}^{(t)}\right]$

where X represents the observed data, Z represents the unobserved data (latent variables or missing data), and θ are the parameters.

Maximization step (M-step): Maximize the expected log-likelihood function obtained from the E-step with respect to the parameters θ. This gives the updated parameter estimates.

Formally

$\hat{\theta}^{(t+1)} = \arg \max_{\theta} Q(\theta \mid \hat{\theta}^{(t)})$

The goal is to find the parameters that maximize the expected log-likelihood.

Iteration: Repeat the E-step and M-step until convergence, i.e., until the parameters $\hat{\theta}^{(t)}$ stabilize and the improvement in the likelihood becomes negligible.

Asymptotic Confidence Bounds

When maximum likelihood estimates (MLEs) do not have a closed form, making it difficult to determine the parameter distribution directly, we rely on the asymptotic distribution of the MLEs to calculate confidence intervals [13]. The asymptotic distribution of the MLE $\widehat{\theta}$ is given by, $\left( \widehat{\theta} - \theta \right) \sim N_{4}(0, I^{-1}(\theta))$ Where I(θ)→ Fisher information matrix of the unknown parameters θ=(α,β,γ,λ).

The elements of the 4 X 4 matrix of I(.), are approximated by 

$I_{ij}\left( \widehat{\theta} \right), \; i,j = 1, 2, 3, 4$

where,

$I_{ij}\left( \widehat{\theta} \right) = \left. -\frac{\partial^{2} l(\theta)}{\partial \theta_{i} \partial \theta_{j}} \right|_{\theta = \widehat{\theta}}$

$\widehat{\theta} = (\widehat{\alpha}, \widehat{\beta}, \widehat{\gamma}, \widehat{\lambda})$ estimated parameters.

Now information matrix can be written as,

$I\left( \widehat{\theta} \right) = - \begin{bmatrix} \frac{\partial^2 l}{\partial \alpha_j^2} & \frac{\partial^2 l}{\partial \alpha_j \partial \beta_j} & \frac{\partial^2 l}{\partial \alpha_j \partial \gamma_j} & \frac{\partial^2 l}{\partial \alpha_j \partial \lambda_j} \\ \frac{\partial^2 l}{\partial \alpha_j \partial \beta_j} & \frac{\partial^2 l}{\partial \beta_j^2} & \frac{\partial^2 l}{\partial \beta_j \partial \gamma_j} & \frac{\partial^2 l}{\partial \beta_j \partial \lambda_j} \\ \frac{\partial^2 l}{\partial \alpha_j \partial \gamma_j} & \frac{\partial^2 l}{\partial \beta_j \partial \gamma_j} & \frac{\partial^2 l}{\partial \gamma_j^2} & \frac{\partial^2 l}{\partial \gamma_j \partial \lambda_j} \\ \frac{\partial^2 l}{\partial \alpha_j \partial \lambda_j} & \frac{\partial^2 l}{\partial \beta_j \partial \lambda_j} & \frac{\partial^2 l}{\partial \gamma_j \partial \lambda_j} & \frac{\partial^2 l}{\partial \lambda_j^2} \end{bmatrix} \tag{7}$

The elements of the fisher information matrix are given in Appendix 1. Therefore, the approximate 100(1-γ)% two-sided, confidence interval for θ is given by $\widehat{\theta} \pm Z_{\gamma/2} \sqrt{I^{-1}\left( \widehat{\theta} \right)} \tag{8}$ Here Z_γ/2_ is the upper γ/2 th percentile of a standard normal distribution.

Information Criterion

The Akaike Information Criterion (AIC) and Bayesian Information Criterion (BIC) are commonly used to assess which of several candidate distributions provides the best fit to the data [13]. A distribution is considered to fit the data well if it has lower AIC and BIC values compared to the others. Both criteria balance goodness of fit with model complexity, penalizing overfitting by including terms related to the number of parameters in the model.

$AIC = 2K - 2\ln L$

$BIC = K\ln(n) - 2\ln L$

Where L is the likelihood function, n is the sample size and K is the number of parameters estimated.

Kaplan-Meier (K-M) Estimator

The Kaplan-Meier estimator of the survival function is a non-parametric method used to estimate the survival function from lifetime data [13]. It is particularly useful for handling censored data, where the exact survival time is unknown for some subjects. The Kaplan-Meier estimator is given by, 

$\widehat{S}(t) = \prod_{i=1}^{n} \left( 1 - \frac{d_{i}}{n_{i}} \right)^{\mathbb{1}_{\{t_{i} &lt; t\}}}$

Where t_i_ is the failure time, d_i_ is the number of events that occurs at time t_i_ and n_i_ is the number individuals at risk of experiencing the event immediately prior to t_i_.

Validation of the model

To validate the modified Weibull model, we analyzed the failure times of patients admitted to BLDE Hospital, Vijayapura. Data were collected from January 1, 2023, to December 31, 2023, comprising 936 patients who passed away during this period.

Table 1 presents the estimated parameters of the model, along with their corresponding standard errors, lower confidence limits (LCL), and upper confidence limits (UCL).

**Table 1:** Estimated values for the real life dataset. UCL: upper confidence limit; LCL: lower confidence limit

Parameter\Causes	Values	Standard Error	LCL	UCL
α	0.001	0.00001377435	0.000973	0.001027
β	1.500999	0.03391981	1.434517	1.567481
γ	1.565522	0.05214207	1.463325	1.667719
λ	0.8138159	0.02118096	0.772302	0.85533

Table 2 provides the estimated AIC and BIC values for various modified forms of the Weibull model. Among all the distributions considered, the newly proposed modified Weibull distribution provided the best fit.

**Table 2:** AIC and BIC values for the data. AIC: Akaike Information Criterion; BIC: Bayesian Information Criterion

Distributions	logl	AIC	BIC
Exponential	-505.0891	1012.178	1017.02
Weibull	-476.1138	956.2276	965.9108
Chen (2000) [5]	-512.9805	1029.961	1039.644
Sarhan and Zaindin (2009) [9]	-476.1168	958.2335	972.7584
Lai et al. (2003) [6]	-476.2299	958.4597	972.6846
Xie et al. (2002) [10]	-470.4547	1012.178	1017.02
New modified Weibull	-468.0408	944.0816	963.448

**Figure 3:**
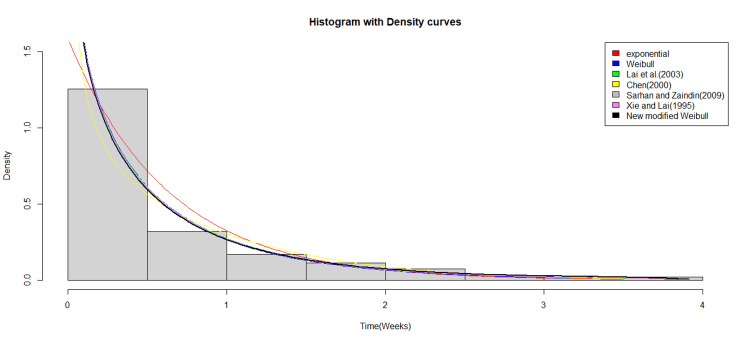
Histogram and fitted density curves for the real life dataset.

**Figure 4:**
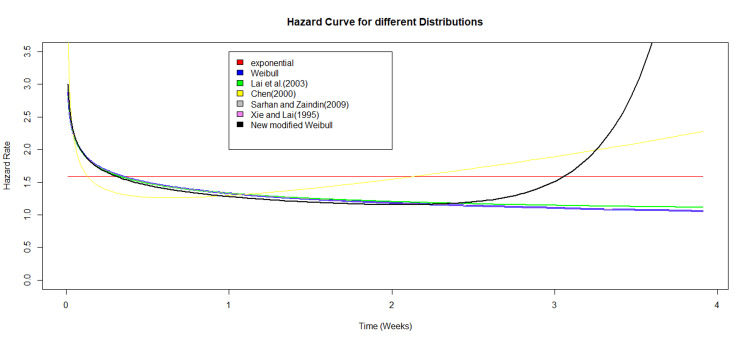
Hazard curves for all distribution using real life dataset.

Figure 3 illustrates the histogram of the data along with the fitted density curves for each distribution. Although Figure 3 may suggest that the data follows an exponential distribution, implying a constant hazard rate, this is not the case. By modifying the distribution, we achieve a more accurate hazard curve, as shown in Figure 4, which is the bathtub.

In Figure 4, the hazard curves for all distributions are presented, with the modified Weibull distribution displaying a characteristic bathtub-shaped hazard function. Initially, the hazard decreases, indicating a reduction in risk. After reaching 0.5 weeks, the hazard stabilizes, maintaining a steady state until about 2.5 weeks, after which the risk begins to increase again.

**Figure 5:**
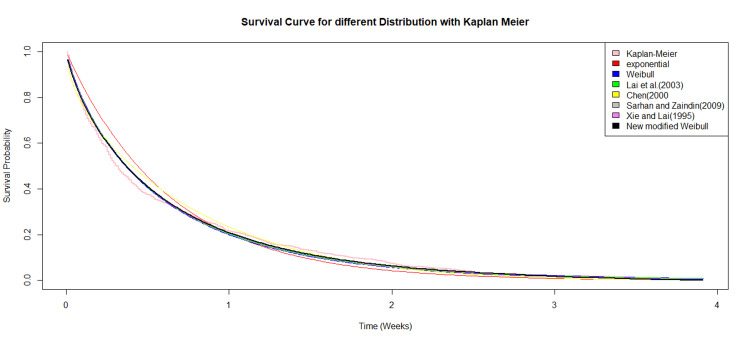
Comparison of Kaplan-Meier survival curve with all distribution survival curves using real life dataset.

Figure 5 compares the survival curves for all distributions, including the Kaplan-Meier survival curve. The modified Weibull distribution provides a better fit to the observed data, with 50% of the patients having died within 0.5 weeks.

## Discussion

The proposed four-parameter modified Weibull distribution is notable for its ability to model complex failure time data characteristics, such as bimodal density and non-monotonic hazard rates. Unlike the three-parameter model introduced by Lai et al. [6], which is less suited for datasets with bimodal behavior and generally shows a decreasing hazard function for the given dataset, the newly introduced model offers greater flexibility. This flexibility is evidenced by its characteristic bathtub-shaped hazard curve (Figure 4), allowing it to more accurately capture a wider range of failure patterns.

Similarly, the four-parameter additive Weibull model proposed by Xie and Lai [10], while more flexible than the three-parameter version, still tends to show a decreasing hazard function, making it less effective in modeling complex patterns such as bimodal data. In contrast, the newly modified Weibull distribution demonstrates superior performance, as reflected in its lower AIC and BIC values (Table 2), indicating a better fit to the data.

Although Figure 3 suggests that the data may follow an exponential distribution, characterized by a constant hazard rate, the modified Weibull model provides a more precise representation of the data. Its key advantage is its versatility, which allows it to represent a wide range of hazard function shapes, including decreasing, constant, increasing, and bathtub-shaped curves. This flexibility makes the model particularly suitable for datasets with intricate failure patterns, such as those exhibiting unimodal or bimodal densities (Figure 1). The model’s ability to capture diverse hazard function shapes, ranging from simple monotonic to more complex bathtub-shaped curves demonstrates its potential in accurately modeling real-world failure time data, whereas traditional models may fall short in capturing the true underlying behavior.

However, a notable limitation of this model is the complexity involved in estimating its parameters. Standard estimation methods may not be effective, requiring simulation techniques to estimate the parameters accurately. Additionally, the moments of this distribution are not directly estimable and also require simulation methods to be computed. This complexity introduces challenges in practical applications, as these simulation-based approaches may be computationally intensive and time-consuming.

## Conclusions

The proposed four-parameter modified Weibull distribution is an effective model for analyzing failure time data, particularly in cases with bimodal density and non-monotonic hazard rates. Its flexibility allows it to capture various hazard shapes, including increasing, decreasing, constant, and, notably, the bathtub-shaped hazard, which is highly relevant in survival analysis.

Validation using hospital records on patient failure times demonstrated a best fit compared to other modified Weibull distributions proposed by different authors. This was confirmed by AIC and BIC values. The estimated standard errors, along with lower and upper confidence limits for the parameters, were provided to ensure the robustness of the parameter estimates.

Additionally, the hazard curves for multiple distributions were plotted, where this model exhibited the characteristic bathtub-shaped hazard. The Kaplan-Meier survival curves were also plotted alongside these distributions for visual comparison of survival functions. The findings suggest that the four-parameter modified Weibull distribution is a flexible and reliable tool for modeling complex survival data with varying hazard rate behaviors, making it a strong candidate for practical applications in survival analysis.

## Appendices

Appendix 1

Using the log likelihood function:

$logL = \sum_{i = 1}^{n}{\log{\left( \frac{\alpha}{\beta^{\alpha}}\ \beta\ t_{i}^{\beta - 1}e^{t_{i}^{\beta}} + \gamma\ \lambda\ t_{i}^{\lambda - 1} \right) -}}\sum_{i = 1}^{n}\left( \frac{\alpha}{\beta^{\alpha}}\left( e^{t_{i}^{\beta}} - 1 \right) + \gamma\ t_{i}^{\lambda} \right)$

$deno = \frac{\alpha}{\beta^{\alpha}}\ \beta\ t_{i}^{\beta - 1}e^{t_{i}^{\beta}} + \gamma\ \lambda\ t_{i}^{\lambda - 1}$

$nume\alpha = \beta\ t_{i}^{\beta - 1}e^{t_{i}^{\beta}}\left( \frac{1 - \alpha\ log\beta}{\beta^{\alpha}} \right)$

$nume\beta = \alpha\frac{\left( t_{i}^{\beta - 1}e^{t_{i}^{\beta}}\left( 2\ \beta\ \log t_{i} + 1 \right) - \alpha\ t_{i}^{\beta - 1}e^{t_{i}^{\beta}} \right)}{\beta^{\alpha}}$

$nume\lambda = \gamma\ t_{i}^{\lambda - 1}\left( \lambda\ logt_{i} + 1 \right)$

The first order partial derivatives with respect to $\alpha, \beta, \gamma, \text{and } \lambda$ were given below.

$\frac{\partial logL}{\partial\alpha} = \sum_{i = 1}^{n}\frac{\beta\ t_{i}^{\beta - 1}e^{t_{i}^{\beta}}\left( \frac{1 - \alpha\ log\beta}{\beta^{\alpha}} \right)}{\frac{\alpha}{\beta^{\alpha}}\ \beta\ t_{i}^{\beta - 1}e^{t_{i}^{\beta}} + \gamma\ \lambda\ t_{i}^{\lambda - 1}} - \sum_{i = 1}^{n}\left( e^{t_{i}^{\beta}} - 1 \right)\left( \frac{1 - \alpha\ log\beta}{\beta^{\alpha}} \right)$

$\frac{\partial logL}{\partial\beta} = \sum_{i = 1}^{n}\frac{\alpha\frac{\left( t_{i}^{\beta - 1}e^{t_{i}^{\beta}}\left( 2\ \beta\ \log t_{i} + 1 \right) - \alpha\ t_{i}^{\beta - 1}e^{t_{i}^{\beta}} \right)}{\beta^{\alpha}}}{\frac{\alpha}{\beta^{\alpha}}\ \beta\ t_{i}^{\beta - 1}e^{t_{i}^{\beta}} + \gamma\ \lambda\ t_{i}^{\lambda - 1}} - \sum_{i = 1}^{n}\left( \frac{\alpha\beta^{\alpha}e^{t_{i}^{\beta}}t_{i}^{\beta}\ logt_{i} - \left( e^{t_{i}^{\beta}} - 1 \right)\ \alpha\ \beta^{\alpha - 1}}{\beta^{2\alpha}} \right)$

$\frac{\partial logL}{\partial\gamma} = \sum_{i = 1}^{n}\frac{\lambda\ t_{i}^{\lambda - 1}}{\frac{\alpha}{\beta^{\alpha}}\ \beta\ t_{i}^{\beta - 1}e^{t_{i}^{\beta}} + \gamma\ \lambda\ t_{i}^{\lambda - 1}} - \sum_{i = 1}^{n}t_{i}^{\lambda}$

$\frac{\partial logL}{\partial\lambda} = \sum_{i = 1}^{n}\frac{\gamma\ t_{i}^{\lambda - 1}\left( \lambda\ logt_{i} + 1 \right)}{\frac{\alpha}{\beta^{\alpha}}\ \beta\ t_{i}^{\beta - 1}e^{t_{i}^{\beta}} + \gamma\ \lambda\ t_{i}^{\lambda - 1}} - \sum_{i = 1}^{n}{\gamma\ t_{i}^{\lambda}}logt$

The second order partial derivatives with respect to

$\alpha, \beta, \gamma and  \lambda$

were given below.

$\frac{\partial^{2}logL}{\partial\alpha^{2}} = \sum_{i = 1}^{n}\frac{\left( deno*\frac{\partial}{\partial\alpha}(nume\alpha) \right) - (nume\alpha)^{2}}{deno^{2}} - \sum_{i = 1}^{n}\left( e^{t_{i}^{\beta}} - 1 \right)\frac{\partial}{\partial\alpha}\left( \left( \frac{1 - \alpha\ log\beta}{\beta^{\alpha}} \right) \right)$

$\frac{\partial^{2}logL}{\partial\beta^{2}} = \sum_{i = 1}^{n}\frac{\left( deno*\frac{\partial}{\partial\beta}(nume\beta) \right) - (nume\beta)^{2}}{deno^{2}} - \sum_{i = 1}^{n}{\frac{\partial}{\partial\ \beta}\left( \frac{\alpha\beta^{\alpha}e^{t_{i}^{\beta}}t_{i}^{\beta}\ logt_{i} - \left( e^{t_{i}^{\beta}} - 1 \right)\ \alpha\ \beta^{\alpha - 1}}{\beta^{2\alpha}} \right)}$

$\frac{\partial^{2}logL}{\partial\gamma^{2}} = \sum_{i = 1}^{n}\frac{\lambda\ t_{i}^{\lambda - 1}}{deno} - \sum_{i = 1}^{n}t_{i}^{\lambda}$

$\frac{\partial^{2}logL}{\partial\lambda^{2}} = \sum_{i = 1}^{n}\frac{\left( deno*\frac{\partial}{\partial\ \lambda}(nume\lambda) \right) - (nume\lambda)^{2}}{deno^{2}} - \sum_{i = 1}^{n}{\lambda\ t_{i}^{\lambda}\left( \log t_{i} \right)^{2}}$

$\frac{\partial^{2}logL}{\partial\alpha\partial\beta} = \frac{\partial^{2}logL}{\partial\beta\partial\alpha} = \sum_{i = 1}^{n}\frac{\left( deno*\ \frac{\partial}{\partial\beta}(nume\alpha) \right) - (nume\alpha\ *nume\beta)}{deno^{2}} - \sum_{i = 1}^{n}{\frac{\partial}{\partial\beta}\left( e^{t_{i}^{\beta}} - 1 \right)}\left( \frac{1 - \alpha\ log\beta}{\beta^{\alpha}} \right)$

$\frac{\partial^{2}logL}{\partial\alpha\partial\gamma} = \frac{\partial^{2}logL}{\partial\gamma\partial\alpha} = - \sum_{i = 1}^{n}\frac{\lambda\ t_{i}^{\lambda - 1}\ nume\alpha}{deno^{2}}$

$\frac{\partial^{2}logL}{\partial\alpha\partial\lambda} = \frac{\partial^{2}logL}{\partial\lambda\partial\alpha} = - \sum_{i = 1}^{n}\frac{nume\lambda*\ nume\alpha}{deno^{2}}$

$\frac{\partial^{2}logL}{\partial\beta\partial\gamma} = \frac{\partial^{2}logL}{\partial\gamma\partial\beta} = - \sum_{i = 1}^{n}\frac{\lambda\ t_{i}^{\lambda - 1}*\ nume\beta}{deno^{2}}$

$\frac{\partial^{2}logL}{\partial\beta\partial\lambda} = \frac{\partial^{2}logL}{\partial\lambda\partial\beta} = - \sum_{i = 1}^{n}\frac{nume\beta*nume\lambda}{deno^{2}}$

$\frac{\partial^{2}logL}{\partial\gamma\partial\lambda} = \frac{\partial^{2}logL}{\partial\lambda\partial\gamma} = \sum_{i = 1}^{n}\frac{\left( deno*t_{i}^{\lambda - 1}*\left( \lambda\ logt_{i} + 1 \right) \right) - \left( \lambda\ t_{i}^{\lambda - 1}*nume\lambda \right)}{deno^{2}} - \sum_{i = 1}^{n}{t_{i}^{\lambda}\ logt_{i}}$
